# Role of Copper and Zinc Ions in the Hydrolytic Degradation of Neurodegeneration-Related Peptides

**DOI:** 10.3390/molecules30020363

**Published:** 2025-01-17

**Authors:** Valentina Pirota, Enrico Monzani, Simone Dell’Acqua, Chiara Bacchella

**Affiliations:** Dipartimento di Chimica, Università di Pavia, Via Taramelli 12, 27100 Pavia, Italy; valentina.pirota@unipv.it (V.P.); enrico.monzani@unipv.it (E.M.)

**Keywords:** peptide hydrolytic degradation, metal ion complexes, LC-MS analysis, neurodegenerative diseases

## Abstract

Spontaneous cleavage reactions normally occur in vivo on amino acid peptide backbones, leading to fragmentation products that can have different physiological roles and toxicity, particularly when the substrate of the hydrolytic processes are neuronal peptides and proteins highly related to neurodegeneration. We report a hydrolytic study performed with the HPLC-MS technique at different temperatures (4 °C and 37 °C) on peptide fragments of different neuronal proteins (amyloid-β, tau, and α-synuclein) in physiological conditions in the presence of Cu^2+^ and Zn^2+^ ions, two metal ions found at millimolar concentrations in amyloid plaques. The coordination of these metal ions with these peptides significantly protects their backbones toward hydrolytic degradation, preserving the entire sequences over two weeks in solution, while the free peptides in the same buffer are fully fragmented after the same or even shorter incubation period. Our data show that peptide cleavage is not only ruled by the chemical sensitivity of amino acids, but the peptide conformation changes induced by metal coordination influence hydrolytic reactions. The enhanced stability of neuronal peptides provided by metal coordination can increase local levels of amyloidogenic species capable of seeding fibril growth, resulting in aberrant protein depositions and deficits in neuronal activity.

## 1. Introduction

Alzheimer’s disease (AD) is one of the most common types of dementia and accounts for 60–70% of all dementia cases worldwide. A breakdown in metal homeostasis and accumulation of protein deposits in neurons are accepted as the prevalent events leading to the onset of this neurological disorder [1,2,3,4,5,6].

In particular, the neurotoxic plaques of β-amyloid peptide (Aβ) predominantly consist of aggregates of peptides with an extension of 39–42 amino acids released upon proteolytic cleavage of the amyloid precursor protein [7]. However, along with the most prevalent forms, many shorter peptides with distinct structural features and toxicities have been detected in amyloid deposits [8].

Although the understanding of the mechanisms and kinetics of amyloid fibril assembly has been recently improved, the multitude of factors involved in the modulation of plaque formation is still partially unknown. In this context, an association between changes in the metal ion concentrations in the neurons and the generation of fibrillary forms of Aβ has been proposed; indeed, transition metal imbalance is tightly associated with oxidative stress, protein aggregation, and synaptic dysfunction in the central nervous system (CNS) [9,10,11]. Both zinc and copper ions are released in the synaptic cleft to modulate the glutamate-mediated neurotransmission by binding to the N-methyl-D-aspartate (NMDA) receptor and the glutamatergic α-amino-3-hydroxy-5-methyl-4-isoxazolepropionic acid (AMPA) receptor [12,13]. Dysregulation of this mechanism can give rise to an aberrant interaction with Aβ peptides since both metals have been found co-localized with the amyloid-β deposits in highly enriched concentrations; moreover, copper (and iron) ions are able to promote the neurotoxic redox activity of Aβ and induce oxidative cross-linking of the peptides into stable oligomers [14,15,16,17,18,19,20]. It is known that metal ions, such as copper(II) but particularly zinc(II), and their complexes can also contribute to peptide bond hydrolysis according to Lewis acid mechanisms [21]. Therefore, the interaction of these metal ions with Aβ may influence in vivo peptide half-life and clearance.

Indeed, one possible strategy for developing new therapeutic drugs for fighting AD decline has been focused on the reduction of Aβ concentration in the brain circulation, particularly targeting its degradation pathway. For example, promotion of the expression levels of some amyloid-degrading proteases (including neprilysin, insulin-degrading enzyme, glutamate carboxypeptidase II, matrix metalloproteinase-9, and others) induces neuroprotection and lowers the predisposition to the development of the disorder [22,23]. In vitro studies showed that smaller-sized Aβ products generated by the enzymatic cleavage were less neurotoxic and more easily cleared [24]. Similar results were obtained after the promotion of Aβ proteolytic degradation mediated by specific antibody fragments [25]. However, some hydrolytic products, i.e., fragments Aβ(25–35) and Aβ(22–35), showed toxicity profiles similar to full-length Aβ monomers [26].

In addition to AD, misfolding processes and imbalances in metal ion homeostasis are highly conserved features across different neurodegenerative pathologies including more than 30 known amyloid-related diseases such as Parkinson’s disease (PD), Huntington’s disease, prion disease, etc. [27]. Therefore, we have also extended our investigation toward other neuronal proteins that directly take part in these processes, such as α-synuclein and tau proteins.

Like Aβ, tau and α-synuclein are prone to give self-propagating aggregation processes which result in the accumulation of insoluble deposits, known as neurofibrillary tangles (NFT, in AD) and Lewy’s bodies (LB, in PD), respectively [28,29]. During the aggregation process, the presence of metal ions, like copper and zinc, actively affects and modulates the stability of these proteins, by inducing post-translational modifications, such as hyperphosphorylation, or conformation changes able to destabilize monomers towards oligomers and fibrils [30,31]. It has been suggested that tau fragmentation contributes to the formation of paired helical filaments (PHF) by increasing aggregation and seeding rates compared to the full-length protein [32,33,34]. However, tau fragmentation is also a normal clearance mechanism that may reduce the severity of this pathology [35,36].

Similarly, α-synuclein hydrolysis catalyzed by proteolytic enzymes is a common process for the clearance of the extracellular excess of this protein [37]. However, this can also lead to the release of toxic fragments that are prone to aggregation and contribute to the progression of PD [38,39].

In general, peptides undergo several degradation pathways through chemical and physical mechanisms. The main chemical processes involve oxidation, hydrolytic cleavage, β-elimination, deamidation, racemization, isomerization, and disulfide bond formation, while changes in the secondary structure of the peptides, resulting in their aggregation and precipitation, are examples of physical modifications. These degradative pathways normally occur in an aqueous solution and are tightly related to critical parameters, such as amino acid sequences, pH, temperature, and the presence of metal cations [40]. Given the importance of assaying the exact role of metal ions when bound to amyloid sequences, we report here how the presence of copper(II) and zinc(II) ions affects peptide hydrolytic degradation over time in physiological buffer conditions.

## 2. Results and Discussion

Herein, we analyzed the hydrolytic process of Aβ peptides in 50 mM Hepes buffer at 7.4 physiological pH over two weeks at two distinct temperatures, 4 °C and 37 °C, using mass spectrometry coupled with high-performance liquid chromatography (HPLC-MS) for precise analysis. Additionally, we explored the impact of copper(II) or zinc(II) ions, each added in a 1:1 molar ratio to the peptides, on the resulting hydrolytic fragmentation patterns. It is worth noting that metal binding only occurs at the *N*-terminal region of Aβ peptides, specifically interacting with histidine residues. The commonly accepted peptide sequence 1–16 (^1^DAEFRHDSGYEVHHNK^16^, Aβ16) is widely used as a model for this interaction due to its inclusion of all three histidines (His6, His13, and His14) known for their key roles in coordinating metal ions [41,42,43,44]. Therefore, the initial studies were performed on this short peptide. Aβ16 has the further advantage of minimizing the aggregation processes that normally occur with longer Aβ peptides. In the latter case, the co-presence of hydrolytic and aggregation/precipitation pathways in the peptide mixtures could preclude the detection and the quantitative estimation of the fragmentation patterns. In this way, the hydrolytic products have been easily identified through *b* and *y* fragments in the MS/MS signals and quantified via their relative percentage areas in the LC-MS chromatograms.

**Figure 1:**
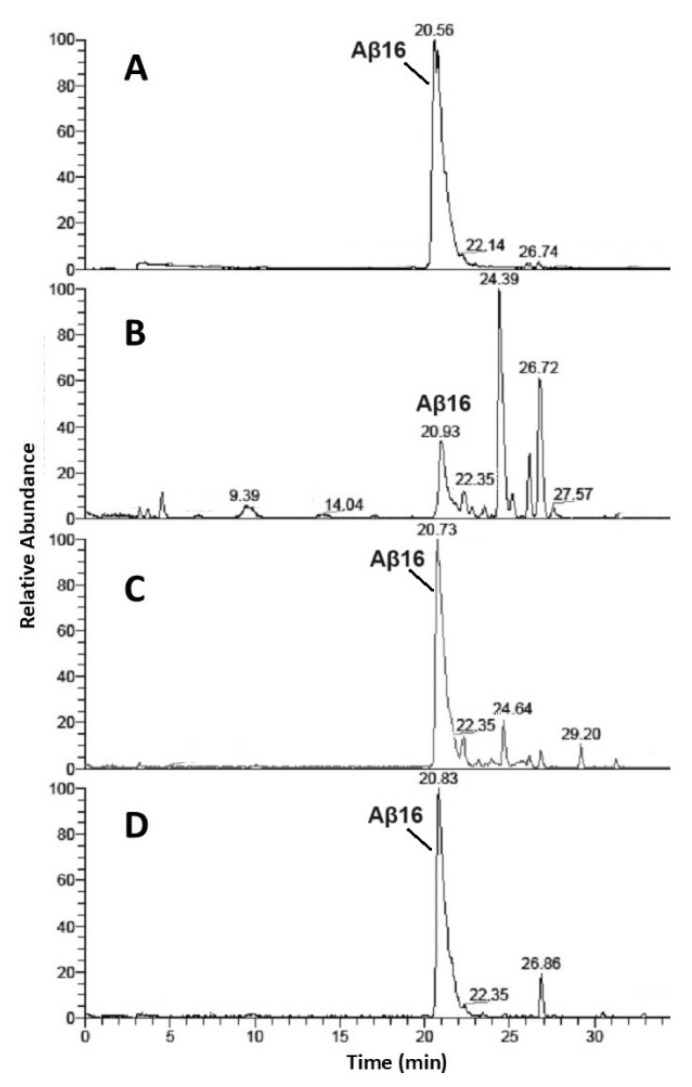
HPLC-MS elution profiles of Aβ16 (500 µM) at time 0 (**A**), after 7 days of incubation time at 37 °C alone (**B**) and with the addition of (**C**) 1 equiv. Cu^2+^ or (**D**) 1 equiv. Zn^2+^ in 50 mM Hepes buffer at pH 7.4. See Table 1 for the assignment of the main peaks at the corresponding retention times.

HPLC-MS analysis showed complete hydrolysis of Aβ16 over two weeks at 37 °C (Figure 1—Table 1), with quite specific cleavages occurring neighboring to aspartate 7 (Asp7, r.t. 9.4 min), glutamate 11 (Glu11, r.t. 26.2 min), and glutamine 15 (Gln 15, r.t. 24.4 min) residues. These findings align with the most common hydrolytic mechanism occurring at the amide bonds in the peptide backbone, often facilitated by acid-catalyzed cleavage mediated by the carboxylic side chains of Asp and Glu. Moreover, peptides containing glutamine (or asparagine) residues are highly susceptible to deamidation which leads, under physiological conditions, to the formation of Glu and Asp, respectively [45].

Interestingly, a comparable amount of fragmentation between the vicinal histidines His13 and His14 was also observed. Although the autoxidation of peptides with molecular oxygen is a relatively slow mechanism, the presence of residues such as Cys, Met, or aromatic amino acids like His, Tyr, and Trp, affects the chemical stability of the peptides, making them more prone to oxidation and further degradation [40].

When the hydrolytic fragmentation of Aβ16 was performed in the presence of copper(II) and zinc(II) ions, a notable stabilization of the peptide was observed. After 7 days of incubation, the peptide integrity percentage increased from 26% in the absence of metals (panel B in Figure 1) to 83% and 90% in the presence of Cu^2+^ and Zn^2+^, respectively (panels C and D in Figure 1). Remarkably, even after 21 days, significant amounts of the native peptide were preserved, approximately 30% with Cu^2+^ and 20% with Zn^2+^ highlighting the protective effect of both metal ions on peptide integrity (Table 1).

**Chart 1:**
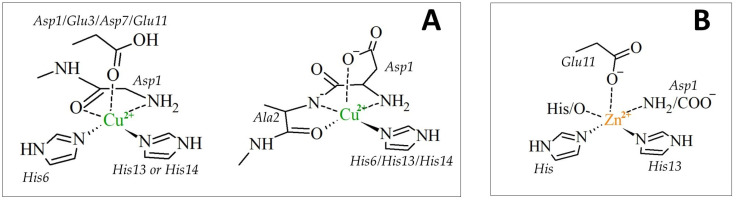
Major forms proposed for copper(II) (**A**) and zinc(II) (**B**) coordination with Aβ peptides at physiological pH [47].

Although the affinity constants of Aβ peptide for copper(II) and zinc(II) differ by four orders of magnitude (10^9^ M^−1^ vs. 10^5^ M^−1^, respectively [46]), and two slightly different coordination spheres have been proposed (Chart 1), their protective activity on the peptide is remarkably similar in both extent and localization.

As described by Butré and coworkers [48], the protection provided by metal coordination does not appear to be uniquely localized near the amino acids acting in their first coordination spheres. This apparent lack of specificity is related to the structural changes of the peptide backbones undergone as a result of the metal ion complexation, which can also alter the internal energy of some peptide bonds, relatively distant from the metal binding site, thus affecting their tendency to be hydrolyzed. Indeed, it was suggested that metal-mediated hydrolytic reactions targeting amino acidic sequences with different extensions, including large proteins such as BSA, occur toward cleavage sites with peculiar local conformations, and are less dependent on their amino acid composition [21].

The same analysis was then repeated at 4 °C to assay the effect of temperature on the hydrolytic stability of the peptide solutions. In these conditions, self-cleavage processes are slower as well as the peptide fibrillar propensity [21,49]. Indeed, the studies at 4 °C not only better control the modification/degradation pathways of the peptide but are also useful to limit aggregation and precipitation processes, commonly associated with longer Aβ species, such as the Aβ28 peptide shown below.

The role of temperature in the structural conversion of the full-length Aβ peptides to fibrillar species has already been elucidated. In vitro experiments provided evidence that thermal transition from 4 °C to 40 °C is able to deeply affect the nucleation and the elongation rates of β-strand structures of the Aβ40 peptide as a result of the promotion of entropy-driven structural transitions and collision frequency [50].

Control experiments on the hydrolytic cleavage of Aβ16 at 4 °C have shown that the peptide fragmentation is strongly reduced if compared to the data collected at 37 °C and, also in the absence of metal ions, the peak of the native peptide is clearly detectable over 21 days (Figure S1). As expected, the main modification sites are generally maintained in the regions flanking the acidic residues and the His-tandem site, although their percent areas are lowered (Table S1). Again, the addition of copper(II) and zinc(II) ions enhances the hydrolytic stability of Aβ16, increasing its percent amounts by approximately 10% after 21 days.

We have then extended our investigations to the study of the hydrolytic stability of the longer Aβ28 peptide (^1^DAEFRHDSGYEVHHQKLVFFAEDVGSNK^28^) in identical conditions. This peptide contains an additional *C*-terminal sequence not involved in metal binding but capable of generating intermolecular interactions to give Aβ fibrillar forms [51]. The hydrolytic fragmentation pattern of Aβ28 in the experiment performed at 4 °C shows higher instability towards degradation over 14 days with respect to shorter peptides and, interestingly, the main cleavage sites are confined in the region encompassing the residue 15–20 (Table S2). This domain has been commonly accepted as the amyloidogenic core of Aβ, but its role is still considered controversial. Indeed, in recent years, the pentapeptide KLVFF has been proposed as a potential anti-aggregation agent for Alzheimer’s disease and it has been identified as directly involved in conformation transition-mediated hydrolytic self-processing [52,53]. This sequence shows a high tendency to undergo self-cleavage from the entire peptides and is capable of acting as an autocatalytic template in the process, thus lowering the availability of toxic full-length Aβ species. Conversely, assemblies of Aβ autocleavage peptides may function as neurotoxins by facilitating the formation of membrane-disrupting pores in neurons [54]. Because of the high tendency to undergo hydrolytic fragmentation of Aβ28, we decided to keep the temperature at 4 °C for the following experiments.

When copper(II) or zinc(II) ions are added to the Aβ28 solutions, the peptide coordination with the metal ions protects from hydrolysis a higher amount of Aβ28 over 21 days, although the protective effect seems to be lower in the case of zinc(II) ions (Figure S2). Moreover, metal coordination does not alter the fragmentation sites but instead reduces the percentage of hydrolytic fragments resulting from the cleavage of the *C*-terminal amyloidogenic core. These results, particularly regarding cleavage localization, differ from data published by our group and others in the presence of copper ions under oxidizing conditions, i.e., with the addition of H_2_O_2_ [55] or reducing substrates like ascorbate or catechols [46,56]. Indeed, during the redox cycling of the metal ion, it was observed that the main cleavage sites corresponded to residues directly involved in or near the metal binding site (for both redox states of copper), primarily restricting fragmentation to *N*-terminal peptide bonds of Asp1/Ala2, Ala2/Glu3, Val12/His13, and His13/His14.

As previously mentioned, Aβ proteolytic peptides have been shown to exhibit both neuroprotective and neurotoxic properties, likely balanced by the tendency of the fragmented species to participate in fibrillation processes. Overall, our findings suggest that metal-mediated resistance to hydrolytic processes could influence Aβ metabolism, resulting in an elevated local concentration of full-length Aβ species within neurons, thereby increasing their vulnerability to Aβ plaque formation. Additionally, the reduced cleavage of the amyloidogenic core may decrease the production of endogenous β-sheet disruptors of fibrillar Aβ deposits, as the KLVFF fragments [57].

Once characterized how copper and zinc influence the hydrolytic process, we extended our attention to other amyloidogenic proteins like tau and α-synuclein by selecting the peptide fragments involved in metal coordination.

**Chart 2:**
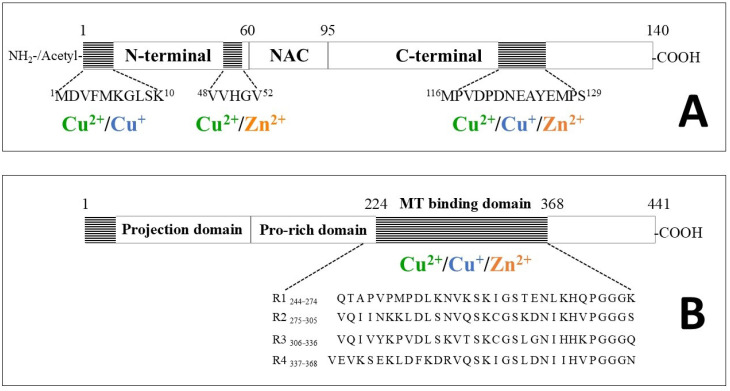
Schematic representation of the structural domains and of the principal metal binding sites in αSyn (**A**) and tau (**B**) protein.

In tau, the binding of copper(II) and zinc(II) ions occurs in a highly-conserved region that shows four pseudo-repeat units (R1–R4), each of them presenting at least one histidine residue [58,59,60,61,62]. Differently, in α-synuclein (αSyn) two binding sites for Cu^2+^ and Zn^2+^ are present: a high-affinity binding site located in correspondence with His50 while a secondary site provided by a carboxylate-rich domain at the *C*-terminus. Moreover, another binding site for copper ion is represented by the *N*-terminus owing to the presence of the terminal amine group (when the protein is not *N*-acetylated) (Chart 2) [63,64,65,66].

To reduce the hydrolytic product population resulting from the cleavage of long amino acid sequences, we have selected two limited regions directly involved in metal binding in tau and αSyn, in particular R1τ (^256^VKSKIGSTENLKHQPGGG^273^) and αSyn15 (^1^MDVFMKGLSKAKEGV^15^), already characterized in terms of metal binding and oxidative (redox) reactivity by our group [63,67,68,69].

Since R1τ corresponds to an internal sequence of the tau protein, the *N*-terminus of the peptide was acetylated, forming Ac-R1τ, to mimic in vivo metal binding provided by this sequence. In contrast, in the case of αSyn, we have synthesized both NH_2_- and Ac- species, to assay the effect of this post-translational modification on the hydrolytic sensitivity of this region in the absence and presence of metal ions.

**Figure 2:**
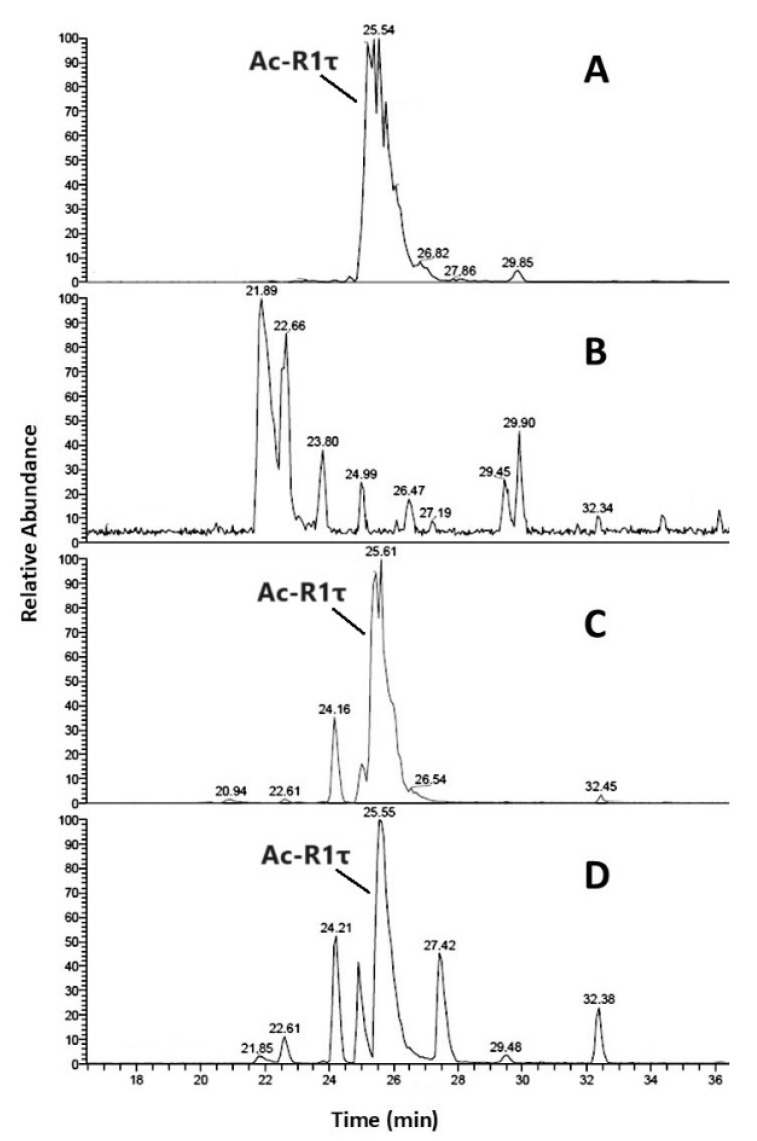
HPLC-MS elution profiles of Ac-R1τ (25 µM) at time 0 (**A**), after 14 days of incubation at 4 °C alone (**B**) and with the addition of (**C**) 1 equiv. Cu^2+^ or (**D**) 1 equiv. Zn^2+^ in 50 mM Hepes buffer at pH 7.4. See Table S3 for the assignment of the main peaks at the corresponding retention times.

**Chart 3:**
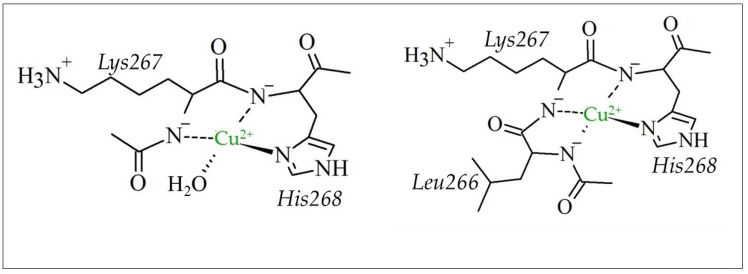
Major forms proposed for copper(II) coordination with Ac-R1τ peptides at physiological pH [69,72].

Ac-R1τ peptide (25 µM) was incubated in 50 mM Hepes buffer at 4 °C for 14 days in the absence and presence of copper(II) or zinc(II) ions in a 1:1 molar ratio with respect to the peptide. The LC-MS analysis carried out on the 14th day of incubation is reported in Figure 2. When the peptide is incubated alone, its hydrolytic degradation quickly occurs, resulting in total cleavage of the peptide after two weeks (Figure 2 panel B—Table S3). Hydrolysis does not occur near specific amino acids, but it rather seems to be generalized to the entire sequence. The addition of copper(II) or zinc(II) ions significantly enhances the stability of Ac-R1τ toward degradative processes, allowing the maintenance of about 85% of the unaltered peptide over 14 days in the presence of copper and approximately 60% if zinc is co-incubated with the peptide (Figure 2C,D). The interaction of zinc(II) to Ac-R1τ has been recently investigated [70], but the coordination mode and the binding affinity have not been defined yet. However, we can assume a similar coordination mode to that found for copper(II) (Chart 3) and predict a higher binding affinity for copper(II) than zinc(II) ions through the Irving–Williams series. The higher protective effect of the former metal ion could likely be associated with a most stable complex with Ac-R1τ [71].

Both metals, on the contrary, direct the peptide cleavage toward a confined region 264–268 (Table S3), nearly suppressing the other hydrolytic sites observed when the peptide is incubated alone.

As stated before, hydrolytic reactions are driven by several factors and do not depend only on the amino acid composition of the peptides. The peptide folding also plays a significant role in directing these reactions. It is therefore likely that the binding of metal ions, occurring in the region neighboring the histidine residue as shown in Chart 3, may induce some structural alterations able to prompt the cleavage mainly toward this region.

**Figure 3:**
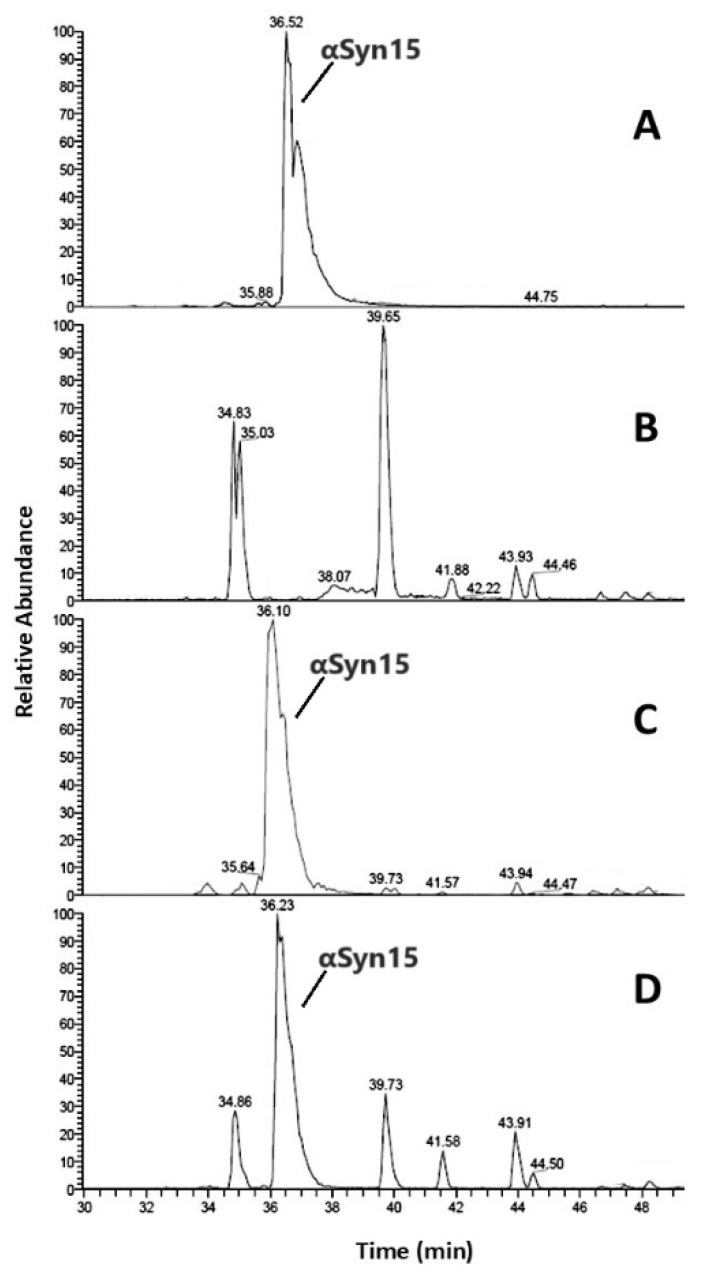
HPLC-MS elution profiles of αSyn15 (25 µM) at time 0 (**A**), after 14 days of incubation at 4 °C alone (**B**) and with the addition of (**C**) 1 equiv. Cu^2+^ or (**D**) 1 equiv. Zn^2+^ in 50 mM Hepes buffer at pH 7.4. See Table S4 for the assignment of the main peaks at the corresponding retention times.

Hydrolytic analysis was then performed on the two isoforms of α-synuclein peptide, αSyn15 and Ac-αSyn15. Like Ac-R1τ, also αSyn15 shows high instability toward hydrolytic degradation, resulting in total cleavage of the amino acidic backbone after 14 days of incubation at 4 °C (Figure 3, panel B).

Acetylation of the *N*-terminal amine group does not significantly affect the extent of the hydrolysis, leading to the complete fragmentation of the peptide upon the same incubation time (Figure S3, panel B). On the other hand, cleavage patterns shown by the two isoforms are quite different (Tables S4 and S5), suggesting that this type of post-translational modification may be able to partially influence the stability of the neighboring peptide bonds. Shifting in fragmentation sites could be related to structural alterations induced by the *N*-protection. It is commonly accepted that the *N*-capping reactions have deep effects on peptide folding [73]. *N*-terminal acetylation of αSyn alters the conformational properties of the *N*-terminal region, resulting in a helicity increase, particularly in the first six residues [74,75]. Moreover, it is worth considering that acetylation of the *N*-terminal primary amine removes one positive charge, thus reducing the net peptide charge from +2 to +1. These changes may affect electrostatic interaction and metal complexation, in particular when the amino acid sequence lacks strong binding ligands, i.e., imidazoles. Furthermore, under the experimental conditions used, the possibility that copper(II) forms a complex with Hepes must be considered, which has a small but non-negligible formation constant (log K = 3) [76]. However, peptides such as Aβ16 (log K = 9) [77], Ac-R1τ (log K = 6.8 [69] and αSyn15 (log K = 10 [78]) have a larger complex formation constant, making the interaction with the buffer negligible. The exception is the peptide Ac-αSyn, where the loss of the *N*-terminal anchor site weakens the coordination of copper, making competition with the Hepes buffer possible.

In agreement with the LC-MS results obtained with the other peptides, over time, the presence of the metal ions protects the entire sequences of the two peptides, particularly reaching percentages of unchanged peptides over 90% in the presence of copper ions after two weeks of incubation. Also in this case, the protection toward peptide degradation provided by zinc ions is lower with respect to copper and this effect is evident when αSyn15 is in the free *N*-terminal form. The role of *N*-acetylation in changing the grade of peptide protection, particularly when zinc is present in solution, was further confirmed by studying a non-physiologically relevant peptide, like R1τ. The data previously obtained with Ac-R1τ were compared to those observed with the *N*-terminally free isoform (Figure S4 and Table S6). After co-incubation for two weeks of the R1τ peptide with zinc ions, the peptide was totally hydrolyzed while, with the *N*-acetylated sequence, the hydrolytic reactions led to the peptide fragmentation with an extent of approximately 40%. We hypothesize the hydrolytic differences mainly reside in different peptide conformations, which are dependent on the chemical properties of the peptides themself and on their metal complexes. Further investigations will be needed to better characterize the nature of these metal/peptide interactions and the folding of metal-bound peptides.

## 3. Materials and Methods

**General.** All chemicals were of reagent grade and purchased from Sigma-Aldrich. Preparative HPLC was carried out with a Shimadzu LC-20AD Prominence instrument equipped with a diode array detector. Mass spectra and LCMS/MS data were performed with an LCQ ADV MAX ion-trap mass spectrometer equipped with an ESI ion source. The instrument was run in an automated LC-MS/MS mode and using a surveyor HPLC system (Thermo Finnigan, San Jose, CA, USA) equipped with a Phenomenex Jupiter 4u Proteo column (4 μm, 150 × 2.0 mm). For the analysis of peptide fragments, Bioworks 3.1 and Xcalibur 2.0.7 SP1 software were used (Thermo Finnigan, San Jose, CA, USA).

**Peptide Synthesis.** The peptides Aβ16 (^1^DAEFRHDSGYEVHHNK^16^-NH_2_), Aβ28 (^1^DAEFRHDSGYEVHHQKLVFFAEDVGSNK^28^-NH_2_), R1τ (^256^VKSKIGSTENLKHQPGGG^273^-NH_2_), Ac-R1τ (Ac-^256^VKSKIGSTENLKHQPGGG^273^-NH_2_), αSyn15 (^1^MDVFMKGLSKAKEGV^15^-NH_2_) and Ac-αSyn15 (Ac-^1^MDVFMKGLSKAKEGV^15^-NH_2_) were synthesized using the standard fluorenyl methoxycarbonyl (Fmoc) solid-phase synthesis in DMF [79,80]. Rink-amide resin MBHA (substitution 0.58 mmol/g or, for Aβ28, 0.26 mmol/g) was used as the solid support, which yielded the peptide amidated at the *C*-terminus. Fmoc deprotection was performed with 20 mL of 20% (*v*/*v*) piperidine in DMF, repeating the reaction twice, for 3 and 7 min. The coupling reactions occurred in the presence of each amino acid (2 mol eqv. vs. resin sites) plus 2 eqv. of N-hydroxybenzotriazole (HOBt), 2 eqv. of benzotriazol-1-yloxytripyrrolidinophosphonium hexafluorophosphate (PyBOP), and ∼2 eqv. of *N*,*N*-diisopropyl ethylamine (DIPEA). The coupling reaction proceeded for at least 45 min and was repeated twice. The capping step was performed by using 20 mL of 4.7% acetic anhydride and 4% pyridine in DMF; the resin was washed with DMF, dichloromethane, and isopropanol. The final cleavage was obtained with a solution of 95% trifluoroacetic acid (TFA, 25 mL for 1 g of resin), triisopropyl silane (2.5%), and water (2.5%). After stirring for 3 h, the peptides were precipitated by adding cold diethyl ether and were filtered. Crude peptides were dissolved in water, for more soluble peptides, or in acidic water (0.1% TFA) for the longer Aβ fragment. They were then purified by HPLC, using a Phenomenex Jupiter 4U Proteo semipreparative column (4 μm, 250 × 10 mm) and a 0−100% linear gradient of 0.1% TFA in water to 0.1% TFA in CH_3_CN over 30 min (flow rate of 3 mL/min, loop 2 mL), as eluent. The identity of the peptides was confirmed by electrospray ionization mass spectrometry (Thermo-Finnigan). ESI-MS data (direct injection, MeOH, positive-ion mode, capillary temperature 200 °C): *m*/*z* 1955 (Aβ_16_H)^+^, 978 (Aβ_16_H_2_)^2+^, 652 (Aβ_16_H_3_)^3+^, 489 (Aβ_16_H_4_)^4+^; 1631 (Aβ_28_H_2_^2+^), 1088 (Aβ_28_H_3_^3+^), 816 (Aβ_28_H_4_^4+^), 653 (Aβ_28_H_5_^5+^); 1879 (Ac-R1τH)^+^, 940 (R1τH_2_)^2+^, 627 (R1τH_3_)^3+^; 1837 (R1τH^+^), 919 (R1τH_2_^2+^), 613 (R1τH_3_^3+^); 1640 (αSyn_15_H^+^), 820.5 (αSyn_15_H_2_^2+^), 547 (αSyn_15_H_3_^3+^), 410 (αSyn_15_H_4_^4+^); 1682 (Ac-αSyn_15_H^+^), 841.5 (Ac-αSyn_15_H_2_^2+^), 561 (Ac-αSyn_15_H_3_^3+^) a.m.u.

**Peptide hydrolysis in the presence of metal ions.** Solutions of Aβ16 and Aβ28 (500 µM) alone or with the addition of Cu(NO_3_)_2_ (1 eqv., 500 µM) or ZnCl_2_ (1 eqv., 500 µM) were prepared in 50 mM Hepes buffer at pH 7.4. The solutions were then incubated at 4 °C or 37 °C (only for Aβ16) in atmospheric air and every 7 days aliquots were taken, diluted to the 25 µM final concentration with Hepes buffer, and analyzed by HPLC-ESI/MS. The solution of R1τ, Ac-R1τ, αSyn15, and Ac-αSyn15 (25 µM) fragments were incubated in 50 mM Hepes buffer at 4 °C for 14 days; at the same time, solutions also containing copper(II) or zinc(II) in a 1:1 molar ratio respect to peptide were prepared. The elution was performed by using 0.1% HCOOH in distilled water (solvent A) and 0.1% HCOOH in acetonitrile (solvent B), with a flow rate of 0.2 mL/min. Elution started with 98% solvent A for 5 min, followed by a linear gradient from 98 to 55% A in 55 min. MS/MS experiments by collision-induced dissociation (CID) were performed with an isolation width of 2 Th (*m*/*z*); the activation amplitude was around 35% of the ejection radiofrequency (RF) amplitude of the instrument. For the analysis of peptide fragments, Bioworks 3.1 and Xcalibur 2.0.7 SP1 software were used (Thermo Finnigan, San Jose, CA, USA).

## 4. Conclusions

The dyshomeostasis of metal ions, such as copper and zinc, is an important pathological feature found in different neurodegenerative diseases characterized by protein misfolding such as AD and PD. In particular, the interaction of copper and zinc ions with amyloidogenic peptides and proteins, including Aβ, tau, and αSyn, is able to modulate their aggregation and toxicity profiles. Indeed, the generation of metal complexes with neuronal peptides and proteins is usually implicated in folding and stability changes connected to post-translational modifications [81,82] and, in the case of copper ions, in the promotion of huge oxidative damage as the product of the redox activity of the metal ions alone or complexed to biological molecules [16,17,18,83,84,85].

In this study, we evaluated how the presence of copper and zinc ions influences the tendency of Aβ peptides of different lengths (Aβ16 and Aβ28), R1 fragments of tau protein, and αSyn15 to undergo hydrolytic degradation.

The data show that all neuronal peptides used in this study, when incubated in solution at physiological pH for two weeks, undergo relatively fast hydrolytic reactions, resulting in their almost complete fragmentation. On the contrary, the presence of the studied metal ions significantly slows down the peptide degradation: the interaction of both copper and zinc ions with the peptides is able to minimize the hydrolysis of the native peptides into smaller fragments for long times, preserving the entire amino acid sequences for more than 14 days.

Moreover, even if the hydrolytic cleavage is an unspecific reaction only marginally ruled by the amino acid sequence, the coordination with metal ions seems to direct the cleavage toward more specific sites because of the structural changes induced by the metal binding. Considering that the main hydrolytic pathways affecting peptide bonds are acid-mediated reactions involving intramolecular rearrangements of the peptide backbones by the side chain carboxyl groups of Asp/Glu, it appears clear that more flexible scaffolds serve as easier substrates of hydrolytic cleavage compared to more organized structures [86].

Indeed, from the studies performed on the insulin peptide by Brange and coworkers, it appeared clear that defined secondary and tertiary structures are able to add conformational stability against peptide cleavage reactions, while the enhancement in structural flexibility in less packed species facilitates deamination and subsequent hydrolysis [87]. In our study, the coordination of the peptides with metal ions can affect the degree of flexibility of the peptides, thus altering the extent of fragmentation. Similarly, the structural changes induced by post-translational modifications, such as the *N*-acetylation, are determinants for peptide fragmentation.

This study thus sets the stage for understanding in greater detail the role of aberrant metal ions (particularly Cu and Zn) in relationship to the metabolism and clearance of Aβ peptides usually found in Alzheimer’s disease brains; the toxicity which may arise from the likely increase in Aβ local levels and higher propensity to generate fibrils needs to be deeper investigated. New studies designed to understand in detail the physiochemical mechanisms involved in the metal-mediated protection against hydrolytic reactions will be performed, particularly by using full-length Aβ species.

## Figures and Tables

**Table 1:** Fragment amount (%) of Aβ16 (500 µM) peptide detected by HPLC-MS analysis, upon incubation in 50 mM Hepes buffer at pH 7.4 at 37 °C alone, and in the presence of 500 µM copper(II) nitrate or 500 µM zinc(II) chloride.

			Aβ16		Cu^2+^-Aβ16			Zn^2+^-Aβ16		
r.t. (min)	Species	a.a.	7 Days	14 Days	7 Days	14 Days	21 Days	7 Days	14 Days	21 Days
9.4	DAEFRHD	1–7	4%	12%	--	--	--	--	--	--
14	SGYEVH	8–13	2%	6%	--	--	--	--	6%	19%
**20.9**	**Aβ16**	**1–16**	**26%**	**--**	**83%**	**63%**	**28%**	**90%**	**78%**	**20%**
22.3	FRHDSGYEVH	4–13	4%	6%	5%	8%	14%	3%	3%	--
23.5	AEFRHDSGYE	2–11	2%	3%	--	--	--	--	--	--
24.4	DAEFRHDSGYEVHHQ	1–15	35%	28%	6%	10%	18%	--	--	--
25.2	DAEFRHDSGYEVHH	1–14	3%	8%	2%	--	2%	--	--	--
26.2	DAEFRHDSGYE	1–11	5%	14%	--	4%	12%	--	--	5%
26.7	DAEFRHDSGYEVH	1–13	17%	19%	2%	8%	13%	7%	13%	51%
27.6	AEFRHDSGYEVHH	2–14	2%	2%	--	--	--	--	--	--
29.2	DAEFRHDSGYEV	1–12	--	2%	2%	7%	13%	--	--	5%

## Data Availability

Data are contained within the article and Supplementary Materials.

## Supplementary Materials

The following supporting information can be downloaded at: https://www.mdpi.com/article/10.3390/molecules30020363/s1. Figure S1. HPLC-MS elution profiles of Aβ16 (500 µM) at time 0 (A), after 21 days of incubation at 4 °C alone (B) and with (C) 1 equiv. Cu^2+^ or (D) 1 equiv. Zn^2+^ in 50 mM Hepes buffer at pH 7.4. See Table S1 for the assignment of the main peaks at the corresponding retention times. Table S1. Detection by LC-MS analysis of the remaining amount (%) of Aβ16 upon incubation alone, and in the presence of 500 µM copper(II) nitrate or 500 µM zinc(II) chloride in Hepes buffer (50 mM) pH 7.4 at 4 °C. Table S2. Detection by LC-MS analysis of the remaining amount (%) of Aβ28 upon incubation alone, and in the presence of 500 µM copper(II) nitrate or 500 µM zinc(II) chloride in 50 mM Hepes buffer at pH 7.4 at 4 °C. Figure S2. HPLC-MS elution profiles of Aβ28 (500 µM) at time 0 (A), after 14 days of incubation at 4 °C alone (B) and with (C) 1 equiv. Cu^2+^ or (D) 1 equiv. Zn^2+^ in 50 mM Hepes buffer at pH 7.4. See Table S2 for the assignment of the main peaks at the corresponding retention times. Table S3. Detection by LC-MS analysis of Ac-R1τ (25 µM) hydrolysis after 14 days of incubation of the peptide alone, and in the presence of 25 µM copper(II) nitrate or 25 µM zinc(II) chloride in 50 mM Hepes buffer at pH 7.4 at 4 °C. Figure S3. HPLC-MS elution profiles of Ac-αSyn15 (25 µM) at time 0 (A), after 14 days of incubation at 4 °C alone (B) and with (C) 1 equiv. Cu^2+^ or (D) 1 equiv. Zn^2+^ in 50 mM Hepes buffer at pH 7.4. See Table S5 for the assignment of the main peaks at the corresponding retention times. Table S4. Detection by LC-MS analysis of αSyn15 (25 µM) hydrolysis after 14 days of incubation of the peptide alone, and in the presence of 25 µM copper(II) nitrate or 25 µM zinc(II) chloride in 50 mM Hepes buffer at pH 7.4 at 4 °C. Table S5. Detection by LC-MS analysis of Ac-αSyn15 (25 µM) hydrolysis after 14 days of incubation of the peptide alone, and in the presence of 25 µM copper(II) nitrate or 25 µM zinc(II) chloride in 50 mM Hepes buffer at pH 7.4 at 4 °C. Figure S4. HPLC-MS elution profiles of R1τ at time 0 (A), after 14 days of incubation at 4 °C alone (B) and with (C) 1 equiv. Cu^2+^ or (D) 1 equiv. Zn^2+^ in 50 mM Hepes buffer at pH 7.4. See Table S6 for the assignment of the main peaks at the corresponding retention times. Table S6. Detection by LC-MS analysis of R1τ (25 µM) hydrolysis after 14 days of incubation of the peptide alone, and in the presence of 25 µM copper(II) nitrate or 25 µM zinc(II) chloride in Hepes buffer (50 mM) pH 7.4 at 4 °C.
