# Supplementary material for: Role of Copper and Zinc Ions in the Hydrolytic Degradation of Neurodegeneration-Related Peptides
Source: Molecules. 2025 Jan 17;30(2):363. doi: 10.3390/molecules30020363 (PMC11767661; doi:10.3390/molecules30020363)
Supplement: Supplementary file 1 [file molecules-30-00363-s001.zip › molecules-3416419-supplementary.pdf]

Supporting Information

Role of Copper and Zinc Ions in the Hydrolytic Degradation of Neurodegeneration-Related Peptides

Valentina Pirota, Enrico Monzani, Simone Dell'Acqua * and Chiara Bacchella *

Dipartimento di Chimica, Università di Pavia, Via Taramelli 12, 27100 Pavia, Italy

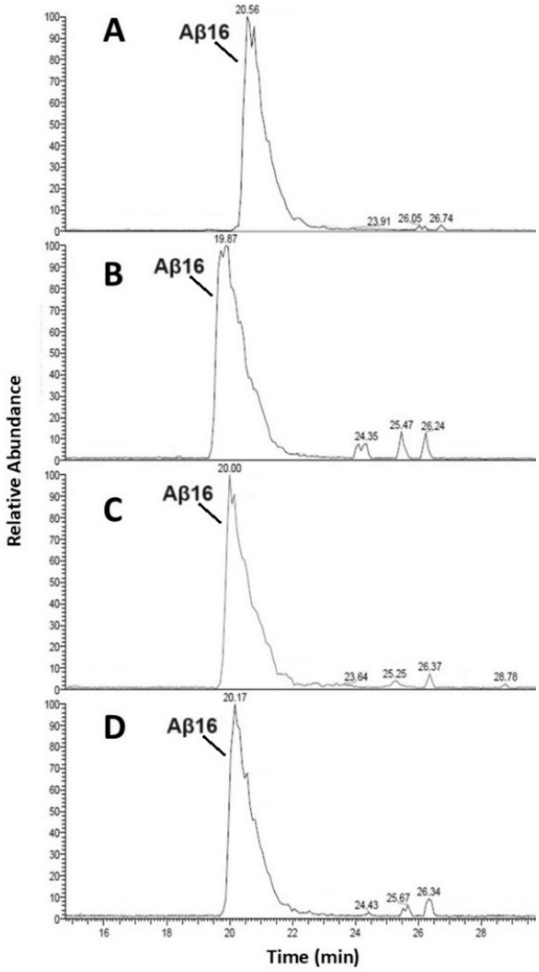

Figure S1 - HPLC-MS elution profiles of Aβ16 (500 μM) at time 0 (A), after 21 days of incubation at 4 °C alone (B) and with (C) 1 equiv. Cu^{2+} or (D) 1 equiv. Zn^{2+} in 50 mM HEPES buffer at pH 7.4. See Table S1 for the assignment of the main peaks at the corresponding retention times.

Table S1 - Detection by LC-MS analysis of the remaining amount (%) of Aβ16 upon incubation alone, and in the presence of 500 μM copper(II) nitrate or 500 μM zinc(II) chloride in HEPES buffer (50 mM) pH 7.4 at 4 °C.

			Aβ16			Cu^{2+}-Aβ16			Zn^{2+}-Aβ16		
r.t. (min)	Species	a.a.	7 days	14 days	21 days	7 days	14 days	21 days	7 days	14 days	21 days
**20.0**	**Aβ16**	**1-16**	**100%**	**90%**	**85%**	**100%**	**100%**	**96%**	**99%**	**97%**	**94%**
24.1	DAEFRHDSGYEVHHQ.K	1-15	-	-	3%	-	-	-	-	-	-
24.5	DAEFRHDSGYEVHH.Q	1-14	-	2%	4%	-	-	-	-	-	1%
25.5	DAEFRHDSGYE.V	1-11	-	2%	5%	-	-	1%	-	-	-
25.7	DAEFRHDSGY.E	1-10	-	-	-	-	-	-	-	1%	2%
26.2	DAEFRHDSGYEVH.H	1-13	-	6%	3%	-	-	2%	1%	1%	3%
28.8	> pyruvate(D)-AEFRH….	1*-16	-	-	-	-	-	1%	-	-	-

Table S2 - Detection by LC-MS analysis of the remaining amount (%) of Aβ28 upon incubation alone, and in the presence of 500 μM copper(II) nitrate or 500 μM zinc(II) chloride in 50 mM HEPES buffer at pH 7.4 at 4 °C.

			Aβ28	Cu^{2+}-Aβ28		Zn^{2+}-Aβ28	
r.t. (min)	Species	a.a.	14 days	14 days	21 days	14 days	21 days
21.2	E.FRHDSGYEVHHQ.K	4-15	2%	-	-	-	-
23.4	F.FAEDVGSNK	20-28	13%	1%	3%	3%	4%
25.5	DAEFRHDSGYEVHHQ.K	1-15	17%	1%	4%	12%	20%
27.4	DAEFRHDSGY.E	1-11	1%	-	-	-	-
27.8	DAEFRHDSGYEVH.H	1-13	3%	-	3%	2%	5%
29.6	DAEFRHDSGYEVHHQKLV.F	1-18	5%	4%	7%	7%	10%
32.0	V.FFAEDVGSNK	19-28	6%	4%	5%	3%	3%
34.3	Q.KLVF.F	16-19	2%	-	-	-	-
35.1	DAEFRHDSGYEVHHQKLVF.F	1-19	-	1%	1%	7%	6%
36.1	L.VFFAEDVGSNK	18-28	5%	1%	1%	4%	6%
**37.1**	**Aβ28**	**1-28**	**0%**	**85%**	**69%**	**35%**	**17%**
38.9	Q.KLVFFAEDVGSNK	16-28	16%	-	-	10%	13%
39.6	DAEFRHDSGYEVHHQKLVFFAE.D	1-22	-	3%	5%	9%	7%
42.5	K.LVFFAEDVGSNK	17-28	22%	1%	1%	5%	4%
43.4	Q.KLVFFAE.D	16-22	1%	-	-	1%	1%
43.7	Q.KLVFFA.E	16-21	-	-	-	-	-
44.7	Q.KLVFF.A	16-20	1%	-	-	-	-
48.5	K.LVFFAE.D	17-22	3%	-	-	1%	4%
49.7	K.LVFFA.E	17-21	1%	-	-	-	-
51.5	K.LVFF.A	17-20	1%	-	-	-	-

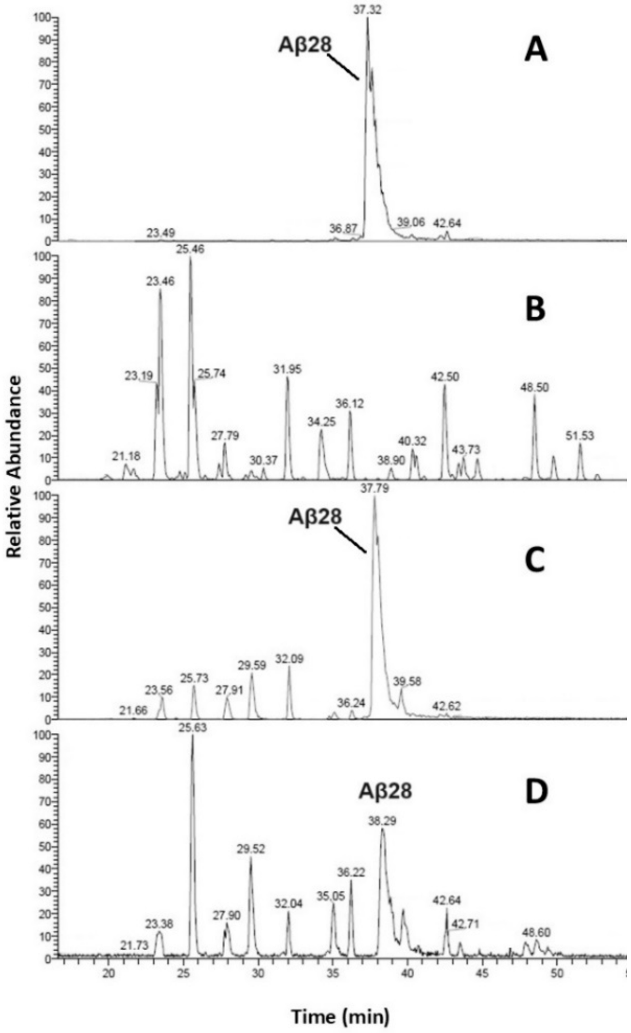

Figure S2 - HPLC-MS elution profiles of Aβ28 (500 μM) at time 0 (A), after 14 days of incubation at 4 °C alone (B) and with (C) 1 equiv. Cu^{2+} or (D) 1 equiv. Zn^{2+} in 50 mM HEPES buffer at pH 7.4. See Table S2 for the assignment of the main peaks at the corresponding retention times.

Table S3 - Detection by LC-MS analysis of Ac-R1τ (25 μM) hydrolysis after 14 days incubation of the peptide alone, and in the presence of 25 μM copper(II) nitrate or 25 μM zinc(II) chloride in 50 mM HEPES buffer at pH 7.4 at 4 °C.

r.t. (min)	Species	a.a.	Ac-R1τ	Cu^{2+}-Ac-R1τ	Zn^{2+}-Ac-R1τ
21.9	K.IGSTENLKHQPGGG	260-273	44%	-	-
22.7	VKSKIG.S	256-261	26%	1%	3%
23.8	VKSKI.G	256-260	8%	-	-
24.2	VKSKIGSTEN.L	256-265	-	11%	14%
25.0	VKSKIGSTE.N	256-264	4%	5%	11%
**25.9**	**Ac-R1t**	**256-273**	**-**	**84%**	**58%**
26.5	S.KIGSTENLKHQ.P	259-269	4%	-	-
27.4	VKSKIGSTENLK.H	256-267	1%	-	15%
29.9	K.IGSTENL.K	260-266	12%	-	-

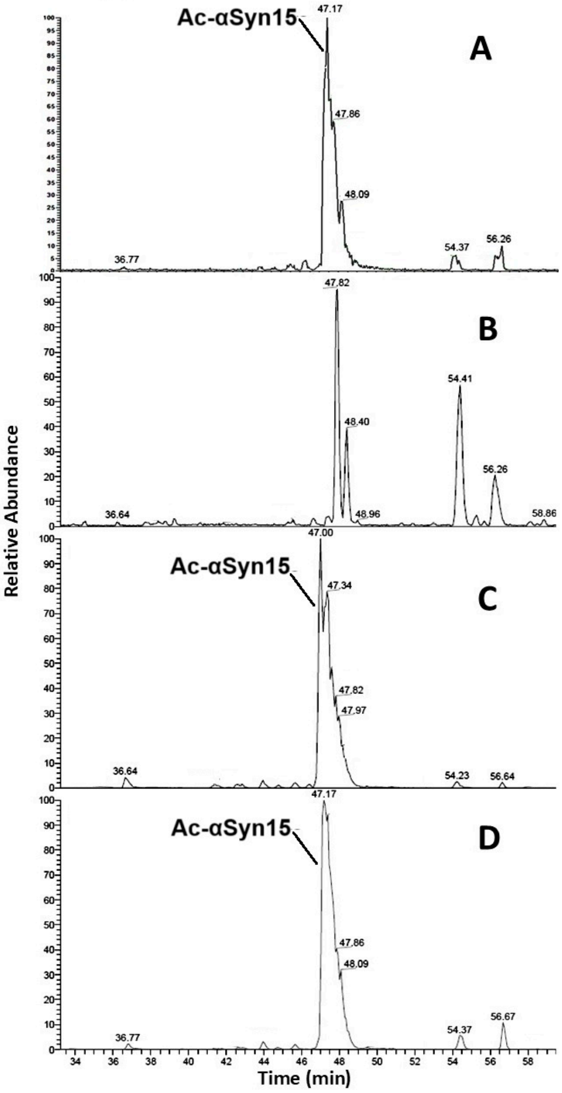

Figure S3 - HPLC-MS elution profiles of Ac-αSyn15 (25 μM) at time 0 (A), after 14 days of incubation at 4 °C alone (B) and with (C) 1 equiv. Cu^{2+} or (D) 1 equiv. Zn^{2+} in 50 mM HEPES buffer at pH 7.4. See Table S5 for the assignment of the main peaks at the corresponding retention times.

Table S4 - Detection by LC-MS analysis of αSyn15 (25 μM) hydrolysis after 14 days incubation of the peptide alone, and in the presence of 25 μM copper(II) nitrate or 25 μM zinc(II) chloride in 50 mM HEPES buffer at pH 7.4 at 4 °C.

r.t. (min)	Species	a.a.	αSyn15	Cu^{2+}-αSyn15	Zn^{2+}-αSyn15
34.0	MDVFMKGLSKAK.E	1-12	-	2%	-
35.1	MDVFMK.G	1-6	38%	1%	10%
**36.6**	**αSyn15**	**1-15**	**-**	**94%**	**68%**
39.6	MDVF.M	1-4	50%	1%	11%
41.6	MDVFMKGLS.K	1-9	3%	-	4%
44.0	MDVFMKGL.S	1-8	8%	1%	8%

Table S5 - Detection by LC-MS analysis of Ac-αSyn15 (25 μM) hydrolysis after 14 days incubation of the peptide alone, and in the presence of 25 μM copper(II) nitrate or 25 μM zinc(II) chloride in 50 mM HEPES buffer at pH 7.4 at 4 °C.

t_R (min)	Species	a.a.	Ac-αSyn15	Cu^{2+}-Ac-αSyn15	Zn^{2+}-Ac-αSyn15
36.6	V.FMKGLSKAKEGV	4-15	-	2%	1%
**47.0**	**Ac-αSyn15**	**1-15**	**-**	**96%**	**94%**
47.9	MDVFMK.G	1-6	40%	-	-
48.4	MDVFMKGLSK.A	1-10	15%	-	-
54.4	MDVFMKGLS.K	1-9	31%	1%	2%
56.3	MDVFMKGL.S	1-8	13%	1%	3%

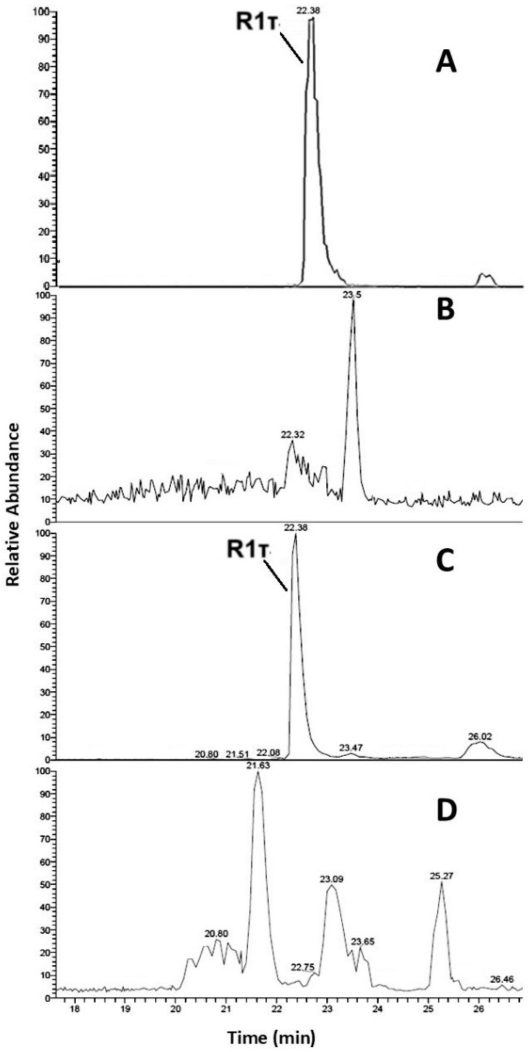

Figure S4 - HPLC-MS elution profiles of R1τ at time 0 (A), after 14 days of incubation at 4 °C alone (B) and with (C) 1 equiv. Cu^{2+} or (D) 1 equiv. Zn^{2+} in 50 mM HEPES buffer at pH 7.4. See Table S6 for the assignment of the main peaks at the corresponding retention times.

Table S6. Detection by LC-MS analysis of R1τ (25 μM) hydrolysis after 14 days incubation of the peptide alone, and in the presence of 25 μM copper(II) nitrate or 25 μM zinc(II) chloride in HEPES buffer (50 mM) pH 7.4 at 4 °C.

r.t. (min)	Species	a.a.	R1τ	Cu^{2+}-R1τ	Zn^{2+}-R1τ
20.8	VKSKIGSTENLK.H	256-267	-	-	15%
21.63	K.IGSTENLKHQPGGG	260-273	-	-	47%
22.32	VKSKIG.S	256-261	46%	-	-
**22.38**	**R1t**	**256-273**	**-**	**82%**	**-**
23.09	VKSKI.G	256-260	-	-	26%
23.52	VKSKIGSTEN.L	256-265	54%	3%	-
25.27	VKSKIGSTE.N	256-264	-	-	12%
26.02	S.KIGSTENLKHQ.P	259-269	-	15%	-
